# Gut microbiota-driven dysbiosis of the SCFA-immune axis in pediatric allergic rhinitis-constipation comorbidity: mechanisms and synbiotic remodeling

**DOI:** 10.3389/fimmu.2025.1639359

**Published:** 2025-12-03

**Authors:** WeiKeng Yang, Xiaojiao Zhang, Bin Wu, Binyu Ni, Hongbin Lin, Congfu Huang

**Affiliations:** 1 The Second Affiliated Hospital, School of Medicine, The Chinese University of Hong Kong, Shenzhen & Longgang District People's Hospital of Shenzhen, Guangdong, China; 2 Child Health Department, Zhuhai Maternal and Child Health Hospital, Zhuhai, China; 3 Department of Pediatrics, Longgang District Maternity & Child Healthcare Hospital of Shenzhen City (Affiliated Shenzhen Women and Children's Hospital (Longgang) of Shantou University Medical College, Medical Research Institute of Maternal and Child, Shenzhen, China

**Keywords:** allergic rhinitis, functional constipation, gut-immune axis, short-chain fatty acids (SCFAs), synbiotic therapy, dysbiosis

## Abstract

**Background:**

The coexistence of allergic rhinitis (AR) and functional constipation (FC) in children reflects complex gut–immune interactions; however, the contribution of microbiota-derived short-chain fatty acids (SCFAs) to immune-metabolic dysregulation remains poorly defined.

**Methods:**

Fecal microbiota from 57 AR-FC children (aged 0–6 years) and 59 age-matched healthy controls (HC) were profiled using 16S rRNA gene sequencing, and functional pathways were inferred via PICRUSt2. A subset of 13 preschoolers (aged 3–7 years) underwent a 3-month synbiotic intervention (multi-strain probiotics combined with dietary fiber), with paired pre- and post-treatment samples analyzed.

**Results:**

AR-FC subjects exhibited reduced α- diversity (*P* = 0.003) and depletion of SCFAs-producing taxa (*Faecalibacterium prausnitzii*: Log2FC = −2.1, *P* = 0.001; *Bacteroides stercoris*: Log2FC = −1.8, *P* = 0.005). Alterations were observed in functional pathways, including upregulated proteasome activity (*P* = 0.01, potentially linked to antigen processing) and suppressed LPS biosynthesis (*P* = 0.02, suggestive of impaired innate immunity). Synbiotic administration enriched *Faecalibacterium* abundance (+54.8%, *P* < 0.05) and alleviated constipation but reduced *Bifidobacterium* (−85.2%, *P* < 0.05), reflecting substrate competition. Following synbiotic intervention, metabolic remodeling was characterized by increased sulfur assimilation (+83.2% sulfate reduction, *P* = 0.04) and diminished β-lactam resistance (−35.4%, *P* = 0.03).

**Conclusion:**

Gut dysbiosis in AR-FC comorbidity is associated with disruption of the microbiota–SCFA–immune axis, which may correlate with mucosal barrier defects and a potential bias toward T helper 2 (Th2) polarization. Although synbiotic therapy induced taxonomic shifts and improved gastrointestinal function, our findings highlight the need for strain-specific formulations to achieve comprehensive immune and intestinal restoration.

## 1 Introduction

The co-occurrence of allergic rhinitis (AR) and functional constipation (FC) in pediatric populations presents a considerable clinical burden, with epidemiological studies reporting a comorbidity prevalence of approximately 20% (1, 2). While gut dysbiosis has been extensively characterized in children with either condition alone (3, 4), the specific microbial and metabolic mechanisms underlying their simultaneous presentation remain inadequately elucidated.

Several research gaps persist. First, most FC-related studies have focused on adult populations (2, 5), neglecting pediatric-specific factors such as age-dependent gut microbial maturation and immune plasticity (6, 7). Second, while taxonomic shifts have been described, functional pathway analyses that elucidate microbiota–metabolite–immune crosstalk are limited (3). Third, although probiotics and prebiotics show promise in managing AR or FC individually (4, 8), their synergistic potential in children with AR-FC comorbidity has not been systematically evaluated.

Emerging evidence highlights the microbiota–short-chain fatty acid (SCFA)–immune axis as a key regulator of mucosal and systemic immunity. SCFAs—particularly butyrate, produced by commensals such as Faecalibacterium prausnitzii and Bacteroides stercoris—enhance epithelial barrier function (6), suppress T helper 2 (Th2) responses via histone deacetylase inhibition (9), and promote regulatory T cell(Treg) differentiation (10). In children, SCFA depletion has been linked to barrier disruption, IgE-mediated sensitization, and Th2/Treg imbalance (9). However, the mechanistic connections among gut dysbiosis, metabolic perturbations, and immune alterations in AR-FC comorbidity remain elusive.

To address these gaps, this study integrates taxonomic, functional, and interventional analyses with clinical phenotypes to investigate gut microbiota-driven immune-metabolic disturbances in pediatric AR-FC. We hypothesize that children with AR-FC exhibit a distinct microbial configuration characterized by depletion of SCFA-producing taxa and disruption of metabolic pathways, contributing to epithelial barrier compromise and Th2-skewed immunity. Our findings aim to advance the microbiota–SCFA–immune axis paradigm and support the development of targeted therapies for pediatric AR-FC comorbidity.

## 2 Methods

### 2.1 Study design and participants

A total of 57 children aged 0–6 years diagnosed with AR-FC and 59 age-matched healthy controls (HC) were recruited from Longgang District Maternity and Child Healthcare Hospital. AR diagnosis followed the established AR guidelines (11), while FC was defined according to the Rome IV criteria (12). Participants were excluded if they: (1) had used antibiotics or probiotics within the past three months; (2) had developmental disorders; or (3) had chronic gastrointestinal diseases.

Sample size estimation was conducted using G*Power 3.1 (13), based on an expected Shannon diversity effect size of 0.8 (α = 0.05, power = 0.8) and anticipated Log_2_FC differences in key taxa (e.g., *Faecalibacterium prausnitzii*, δ = −2.0, σ = 1.2).

Written informed consent was obtained from guardians. The study protocol was approved by the Institutional Review Board of Longgang District Maternity and Child Healthcare Hospital (Approval No. KYXMLL-01-CZGC-14-2-1). The trial was registered in the Chinese Clinical Trial Registry (Registration number: ChiCTR2400085982; Reg Date:2024-06-21).

### 2.2 Participant characteristics

Demographic and clinical characteristics of the AR-FC and HC groups are presented in **Table 1**. The two groups were comparable in terms of age, sex, height, and weight. No significant differences were observed in delivery mode, feeding history, orneonatal intensive care unit (NICU) admission history (all *P* > 0.05).

**Table 1:** Demographic and clinical characteristics of the AR-FC and healthy control (HC) groups.

Characteristic	AR-FC (n=57)	HC (n=59)	Statistical test	*P*-value
Age (year), mean ± SD	4.8 ± 0.9	4.7 ± 1.0	t-test	0.603
Sex (Male), n (%)	32 (56.1%)	35 (59.3%)	χ² test	0.669
Height (cm), mean ± SD	108.5 ± 6.2	107.8 ± 5.9	t-test	0.532
Weight (kg), mean ± SD	19.2 ± 2.7	18.9 ± 2.5	t-test	0.543
Delivery mode (Vaginal), n (%)	41 (71.9%)	44 (74.6%)	χ² test	0.744
Exclusive breastfeeding (≥6 mo), n (%)	36 (63.2%)	38 (64.4%)	χ² test	0.892
History of NICU admission, n (%)	8 (14.0%)	6 (10.2%)	χ² test	0.522

### 2.3 Fecal sample processing and *16S rRNA* gene sequencing

Fecal samples (~5g) were collected in sterile containers, immediately flash-frozen at −80 °C, and transported to BGI Precision Nutrition Co., Ltd for analysis. Total genomic DNA was extracted using the PowerSoil^®^ DNA Isolation Kit (MoBio Laboratories, USA) following the manufacturer’s protocol.

The V3–V4 hypervariable regions of the 16S rRNA gene were amplified using primers 338F (5′-ACTCCTACGGGAGGCAGCAG-3′) and 806R (5′-GGACTACHVGGGTWTCTAAT-3′). Polymerase chain reaction (PCR) amplification was performed under the following conditions: initial denaturation at 94 °C for 5 min; 30 cycles of denaturation at 94 °C for 30 s, annealing at 52 °C for 30 s, and extension at 72 °C for 45 s; followed by a final extension at 72 °C for 10 min. Purified amplicons were quantified and pooled in equimolar concentrations, then sequenced on the Illumina MiSeq platform (2 × 250 bp paired-end reads).

### 2.4 Synbiotic intervention protocol

Thirteen preschool-aged children with AR-FC (aged 3–7 years) received oral administration of a multi-strain probiotic formulation (3 g/sachet containing 1×10^10^ CFU viable bacteria per sachet, comprising *Lactobacillus paracasei GM080, L. paracasei LT12, L. acidophilus DDS-1, L. rhamnosus UAlr-06, L. fermentum GM090*, and *Bifidobacterium lactis UABla-12*) combined with a dietary fiber supplement (3 g/sachet providing 2.6 g fiber from partially hydrolyzed guar gum, resistant dextrin, and fructooligosaccharides). The initial dosage was one sachet twice daily, which was reduced to once daily after two weeks or upon significant constipation relief. Paired fecal samples were collected before and after the 3-month intervention.

### 2.5 Bioinformatic and statistical analyses

Raw sequencing reads were subjected to quality control using Trimmomatic v0.39, wherein reads with Phred quality scores (Q-score) < 30, ambiguous bases, or lengths < 200 bp were discarded. Chimeric sequences were identified and removed using USEARCH v11.0.667, implementing the *de novo* UCHIME algorithm. High-quality paired-end reads were subsequently merged with FLASH v1.2.11, and operational taxonomic units (OTUs) were clustered at a 97% sequence similarity threshold using USEARCH. Taxonomic annotation was performed against the Greengenes database (v2013).

α- diversity indices (Shannon index) and β-diversity metrics (Bray–Curtis dissimilarity) were computed in R v3.3.3 using the “ade4” package. Differential taxonomic abundance between groups was assessed via Wilcoxon rank-sum tests with Benjamini–Hochberg false discovery rate (FDR) correction (adjusted *P* < 0.05). Functional prediction analysis was inferred using PICRUSt2 v2.4.2 based on Kyoto Encyclopedia of Genes and Genomes (KEGG) Orthology (KO) annotations.

## 3 Results before intervention

### 3.1 Gut microbiota diversity, composition, and taxonomic alterations in children with coexisting allergic rhinitis and functional constipation

**Figure 1:**
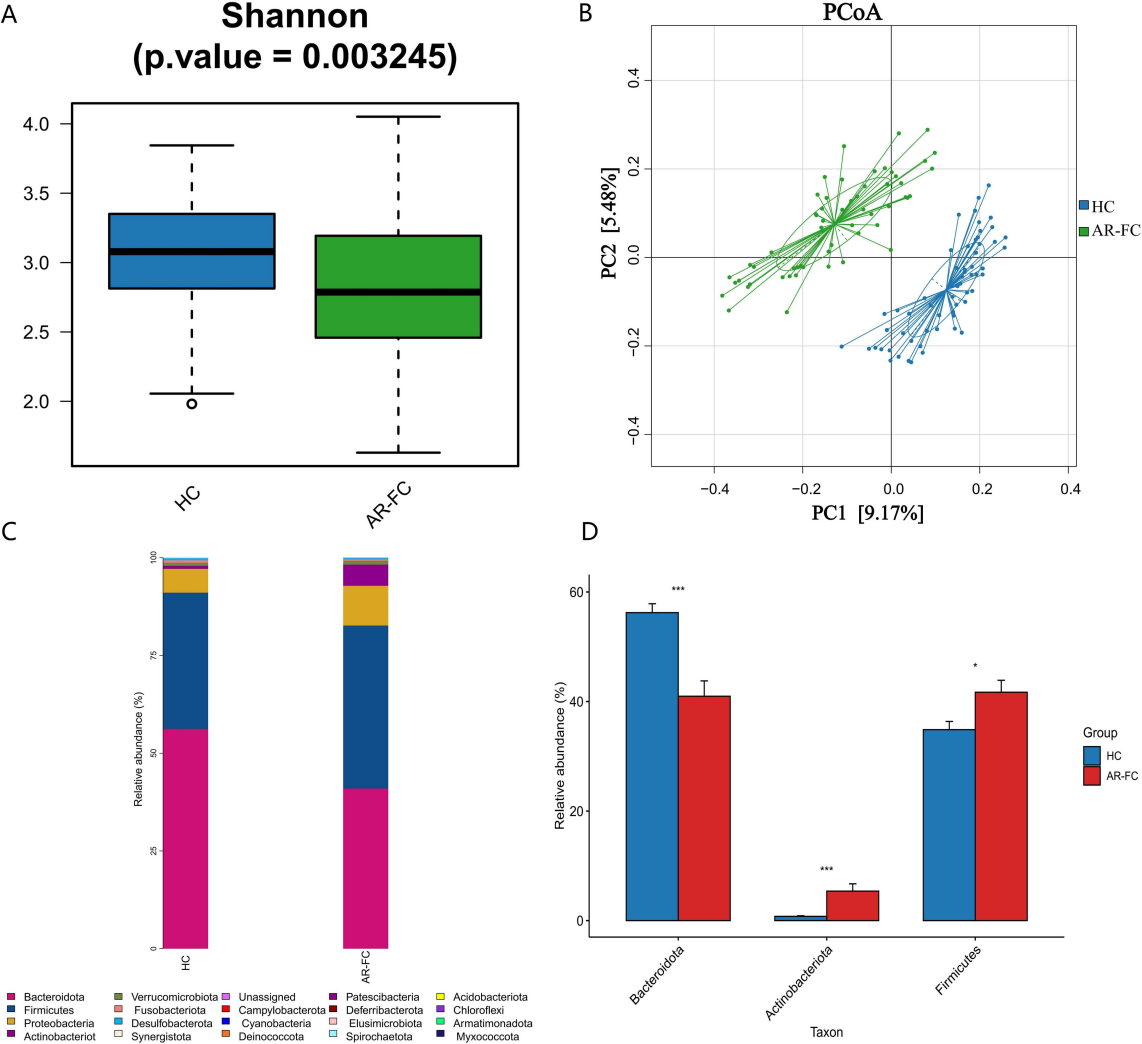
Gut microbiota diversity and phylum-level composition between AR-FC and HC groups. **(A)** α-diversity analysis (Shannon index) showing reduced microbial diversity in AR-FC children (Wilcoxon rank-sum test, *P* = 0.003). **(B)** Principal coordinates analysis (PCoA) of β-diversity based on Bray-Curtis dissimilarity (PERMANOVA, *P* = 0.002), showing significant separation between AR-FC and HC groups. **(C)** Relative abundance of dominant phyla (*Bacteroidetes, Firmicutes, Actinobacteria, Proteobacteria*, and *Fusobacteria*). **(D)** Differential phylum abundance between groups (*P* < 0.05; *P* < 0.01; *P* < 0.001; Wilcoxon test with FDR correction). AR-FC, Allergic rhinitis with functional constipation; HC, Healthy control.

Comparative analysis revealed marked gut microbial dysbiosis in children with AR-FC compared to HCs. α-diversity, assessed by the Shannon index, was significantly reduced in the AR-FC group (*P* = 0.003; **Figure 1A**), while β-diversity analysis using principal coordinates analysis (PCoA) revealed a clear separation between the AR-FC and HC groups (PERMANOVA, P = 0.002; **Figure 1B**), indicating significant differences in microbial community structure.

**Figure 2:**
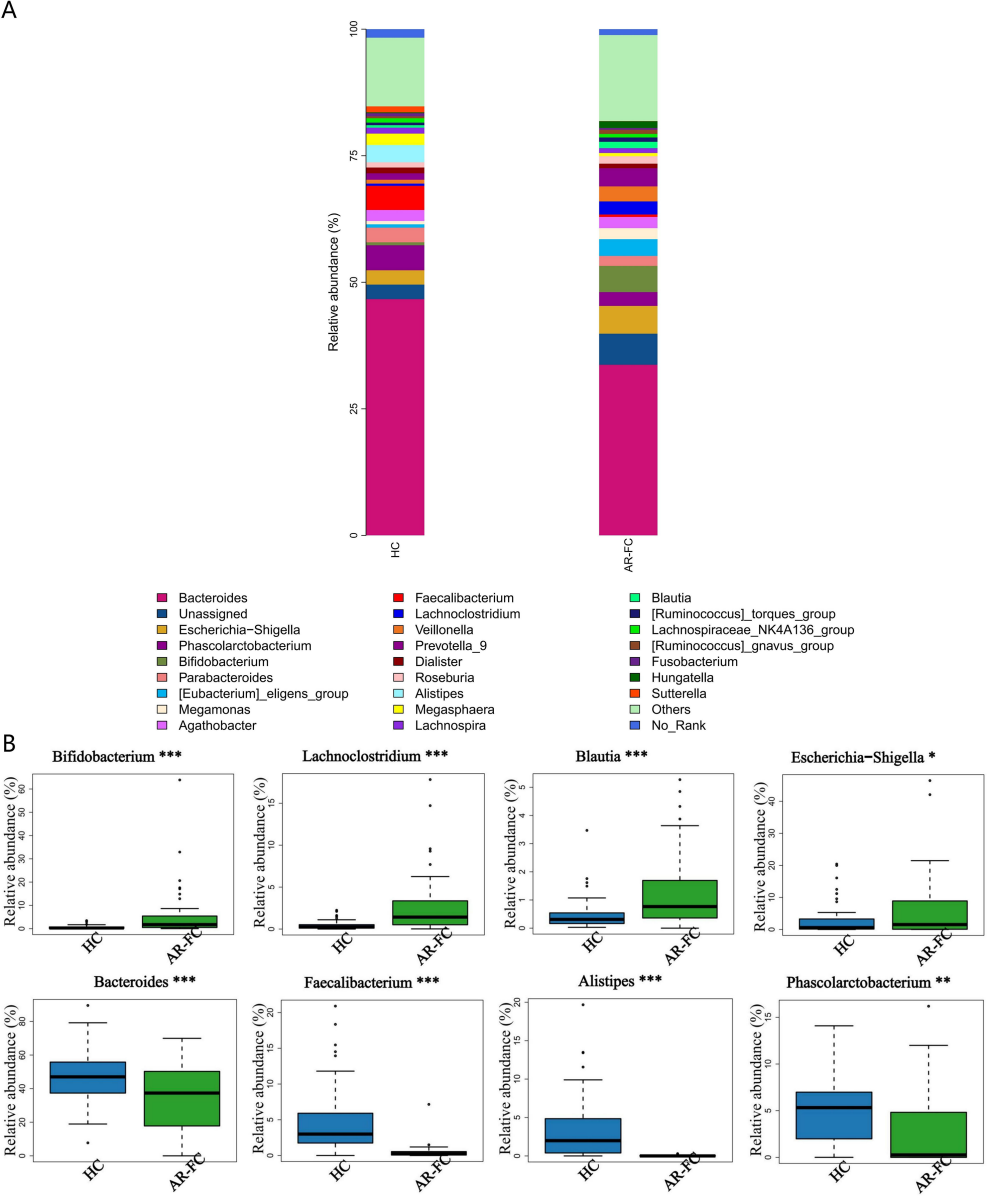
Genus-level taxonomic differences in gut microbiota. **(A)** Stacked bar plot showing the relative abundance of dominant genera. **(B)** Differentially abundant genera between AR-FC and HC groups. Key findings: *Bifidobacterium, Lachnoclostridium, Blautia*, and *Erysipelatoclostridium* were enriched in AR-FC, while *Bacteroides*, *Faecalibacterium, Alistipes*, and *Phascolarctobacterium* were depleted (*P < 0.05, **P < 0.01, ***P < 0.001; Wilcoxon test with FDR correction).

This dysbiosis was associated with pronounced taxonomic alterations across multiple hierarchical levels. At the phylum level, the relative abundance of Bacteroidetes was markedly decreased (Log_2_FC = −1.5, *P* = 0.003), whereas Firmicutes and Actinobacteria were significantly enriched (*P* < 0.05; **Figures 1C, D**). Genus-level profiling revealed enrichment of *Bifidobacterium*, *Lachnoclostridium*, *Blautia*, and *Erysipelatoclostridium* in AR-FC, concomitant with depletion of *Bacteroides*, *Faecalibacterium*, *Alistipes*, and *Phascolarctobacterium* (all *P* < 0.05; **Figures 2A, B**).

**Figure 3:**
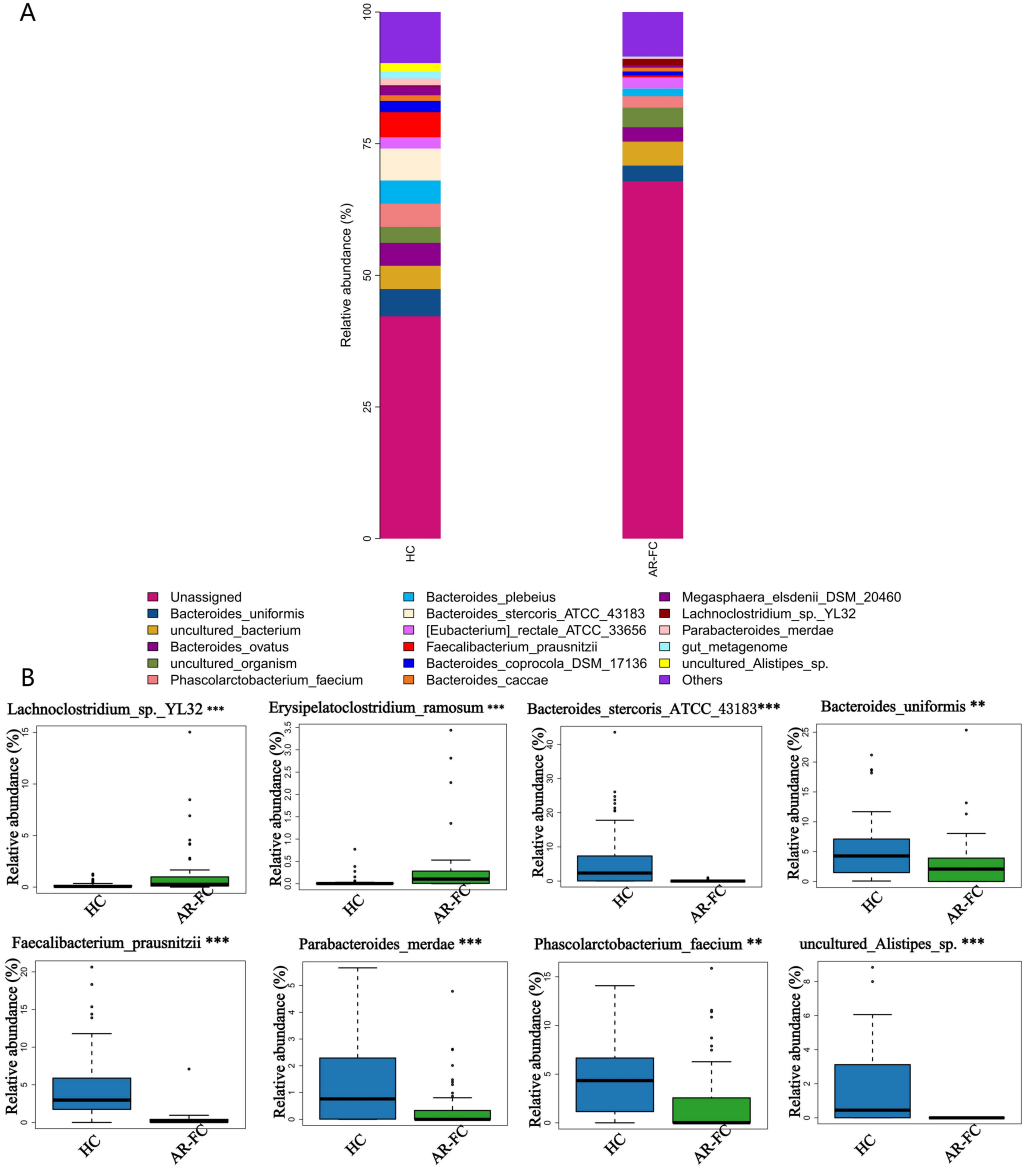
Species-level microbial signatures of AR-FC comorbidity. **(A)** Heatmap of dominant species abundance. **(B)** Differentially abundant species, including reduced Bacteroides stercoris (Log2FC=-1.8, P = 0.005) and Faecalibacterium prausnitzii (Log2FC = -2.1, *P* = 0.001) in AR-FC (**P < 0.01, ***P < 0.001; Wilcoxon test with FDR correction).

At the species level, the most pronounced reductions were observed in key SCFA-producing taxa, including *Faecalibacterium prausnitzii* (Log_2_FC = −2.1, *P* = 0.001) and *Bacteroides stercoris* (Log_2_FC = −1.8, *P* = 0.005; **Figures 3A, B**). A comprehensive summary of these differentially abundant taxa is presented in **Table 2**.

**Table 2:** Differentially abundant taxa in AR-FC *vs.* HC groups.

Taxonomic level	Taxon	AR-FC vs. HC (Log_2_FC)	*P*-value (FDR-adjusted)
Phylum	Bacteroidetes	−1.5	0.003
Phylum	Firmicutes	+1.2	0.012
Phylum	Actinobacteria	+0.9	0.028
Genus	*Faecalibacterium*	−2.1	0.001
Genus	*Bacteroides*	−1.7	0.004
Genus	*Bifidobacterium*	+1.5	0.018
Genus	*Lachnoclostridium*	+1.3	0.022
Species	*Faecalibacterium prausnitzii*	−2.1	0.001
Species	*Bacteroides stercoris*	−1.8	0.005
Species	*Erysipelatoclostridium ramosum*	+1.6	0.016

### 3.2 Functional prediction analysis reveals altered metabolic pathways

**Figure 4:**
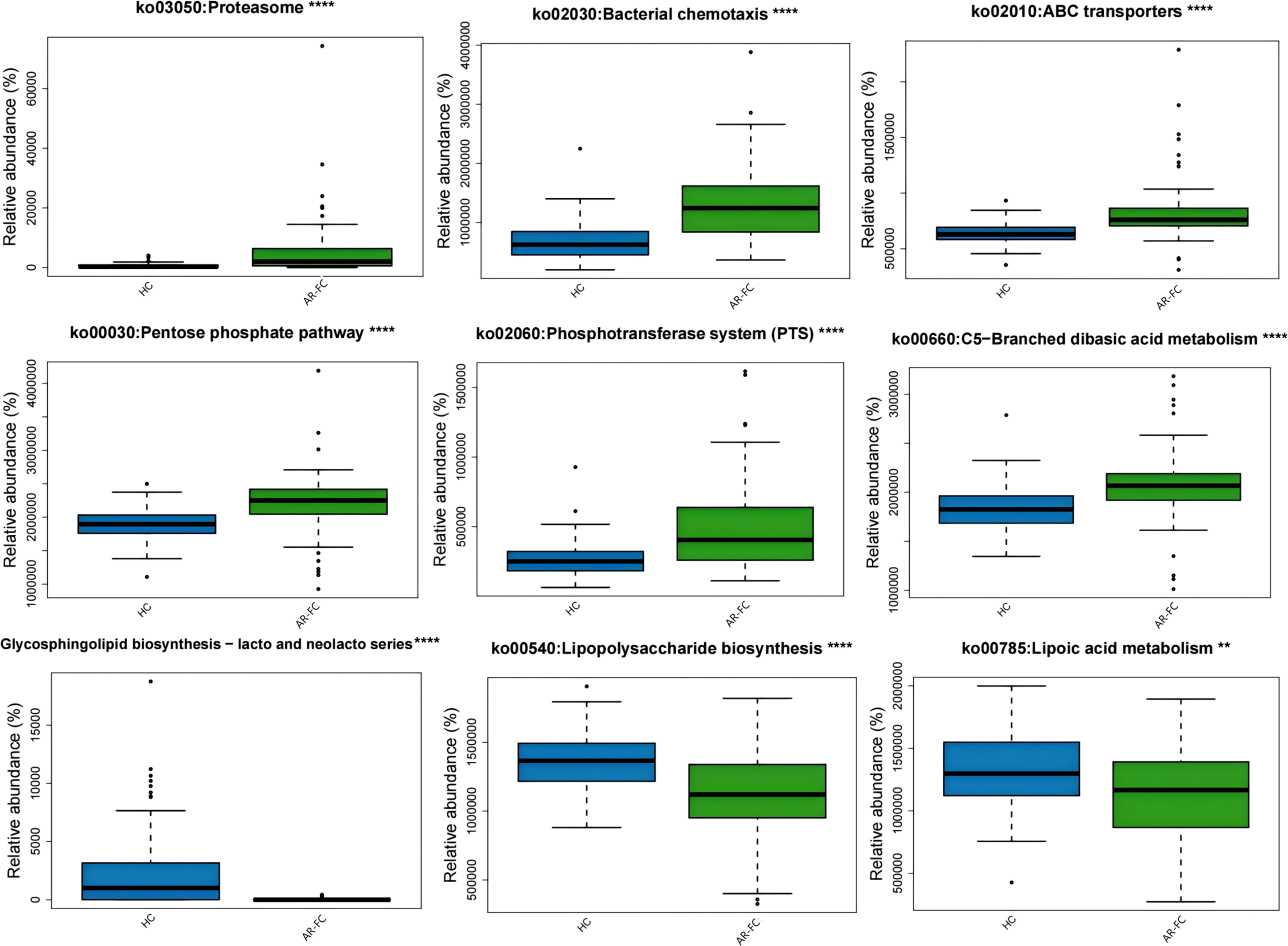
Functional pathway alterations predicted using PICRUSt2 based on KEGG Orthology annotations. The AR-FC group showed upregulated proteasome pathways (P = 0.01) and downregulated lipopolysaccharide biosynthesis (P = 0.02) ( **P < 0.01, ****P < 0.0001; Wilcoxon test with FDR correction). Key pathways included enrichment of proteasome, bacterial chemotaxis, and ABC transporters and depletion of glycosphingolipid biosynthesis and lipoic acid metabolism(Linear discriminant analysis [LDA] > 2.0, P < 0.05).

Functional prediction analysis revealed significant perturbation in microbial metabolic functions in the AR-FC group compared to HCs. Pathways related to proteasome activity (*P* = 0.01), bacterial chemotaxis (*P* = 0.02), and ABC transporters (*P* = 0.03) were upregulated. In contrast, lipopolysaccharide (LPS) biosynthesis (*P* = 0.02) and glycosphingolipid metabolism (*P* = 0.01) were suppressed (**Figure 4**). Functional prediction analysis also revealed a notable finding: despite the marked depletion of key SCFA-producing taxa, KEGG pathways directly involved in SCFA biosynthesis, including propanoate metabolism (FDR-adjusted *P* < 0.01) and butanoate metabolism (FDR-adjusted *P* < 0.01), were significantly upregulated in the AR-FC group compared to HCs (**Supplementary Figure S1**). This apparent discrepancy between taxonomic depletion and functional pathway enrichment may suggest compensatory upregulation of SCFA metabolic pathways in residual microbial populations, or alternatively, a dysregulated state where pathway transcription is decoupled from actual metabolite output.

## 4 Post-intervention outcomes

### 4.1 Synbiotic intervention restores microbial balance

**Figure 5:**
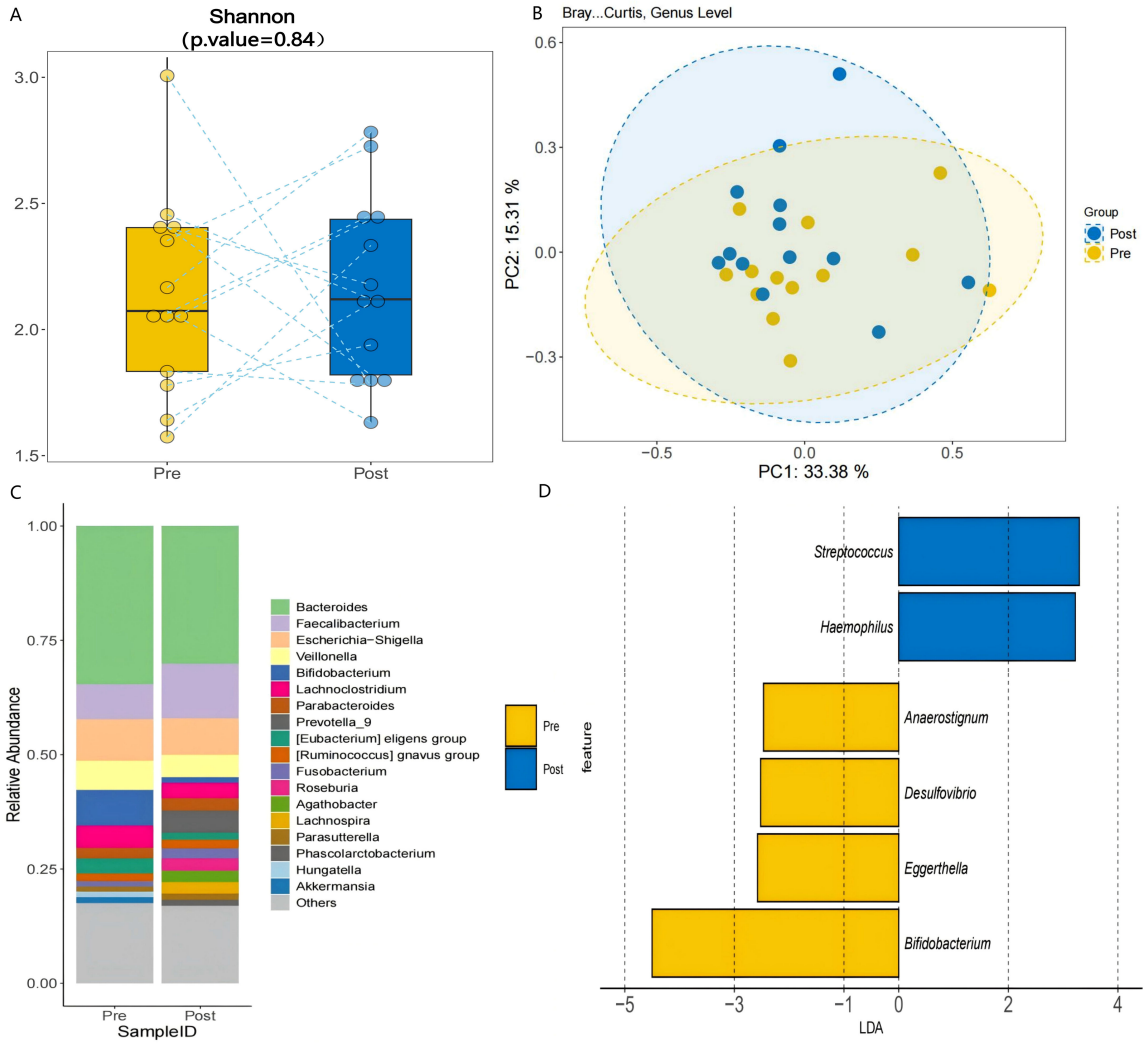
Post-intervention microbial changes. **(A)** α-diversity comparison pre- *vs.* post-intervention (Shannon index, *P* = 0.84). **(B)** PCoA illustrating structural shifts in microbial communities pre- and post-intervention (PERMANOVA, P = 0.12; PC1: 33.38% variance). **(C)** Genus-level abundance changes: *Faecalibacterium* increased by 54.8% (*P* < 0.05), and *Bifidobacterium* decreased (*P* < 0.05). **(D)** LDA analysis identified *Faecalibacterium* as a discriminant taxon post-intervention (LDA > 2.0, *P* < 0.05), consistent with its increased relative abundance.

A 3-month synbiotic intervention (probiotics + dietary fiber) in 13 AR-FC children induced taxonomic shifts in the gut microbiota, though β-diversity changes were not statistically significant (PERMANOVA, P = 0.12; PC1: 33.38% variance; **Figure 5B**). The intervention notably increased the relative abundance of *Faecalibacterium* by 54.8% (*P* < 0.05), whereas *Bifidobacterium* levels decreased by 85.2% (*P* < 0.05; **Figures 5C–D**, **Supplementary Table S1**). A reduction of 12.3% was also observed in *Escherichia–Shigella*, a known pathobiont, though this change did not reach statistical significance.

### 4.2 Functional reprogramming following synbiotic treatment

**Figure 6:**
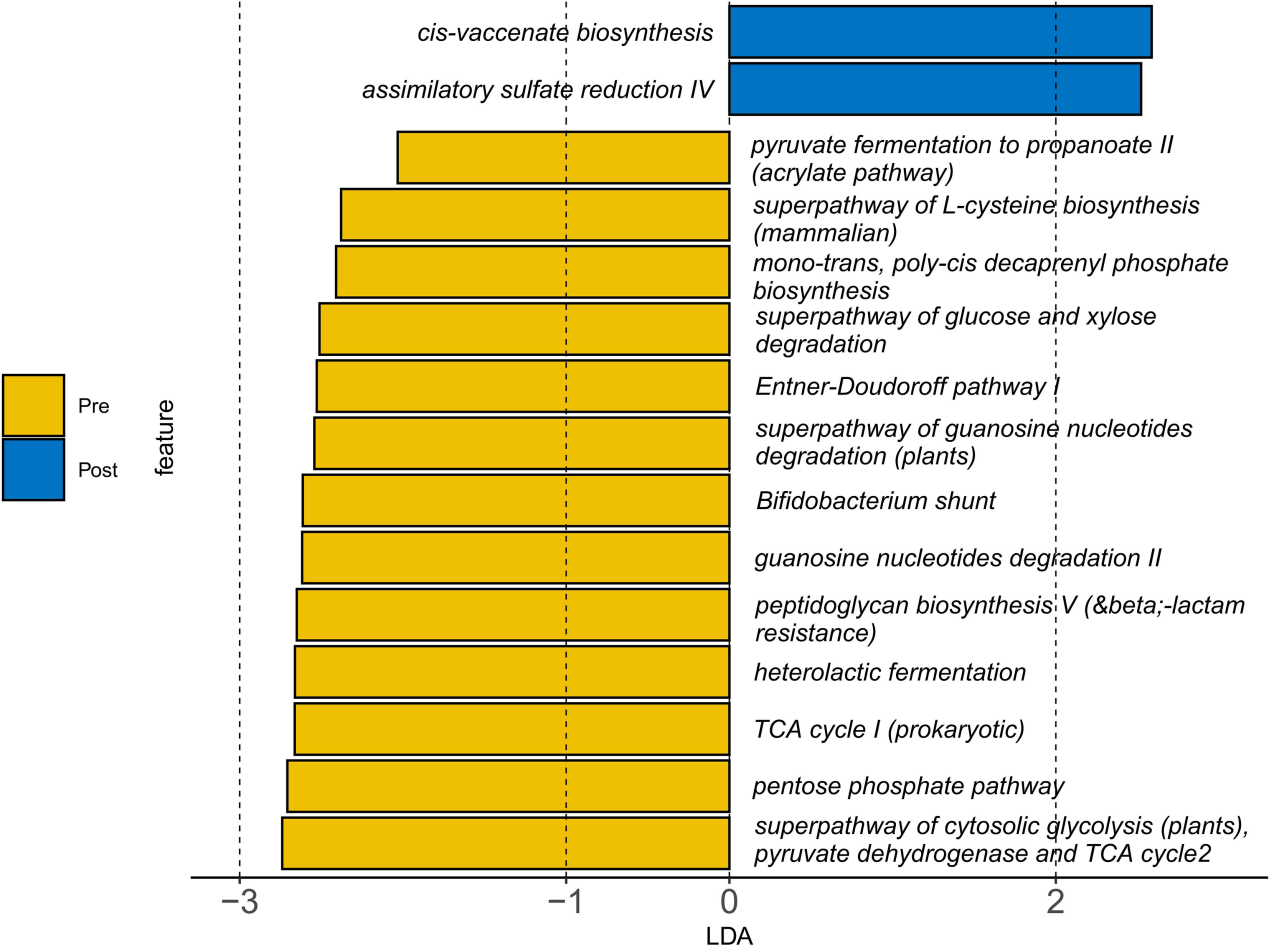
Changes in gut microbial metabolic pathways following synbiotic intervention. Functional prediction (PICRUSt2) illustrate the comparison between pre- and post-intervention states. The results show a shift from pre-intervention enrichment in carbohydrate metabolism pathways (e.g., heterolactic fermentation) toward post-intervention upregulation of lipid and sulfur metabolism (e.g., cis-vaccenate biosynthesis and assimilatory sulfate reduction). A reduction in β-lactam resistance pathways was also observed post-intervention. Statistical significance was determined by Linear Discriminant Analysis (LDA) score > 2.0 and P < 0.05.

Functional prediction analysis indicated a metabolic shift from carbohydrate-dominant pathways (e.g., heterolactic fermentation, *P* = 0.008) toward enhanced lipid and sulfur metabolism after intervention. Specifically, Cis-vaccenate biosynthesis increased by 11.6% (*P* = 0.03), and assimilatory sulfate reduction rose by 83.2% (*P* = 0.04; **Figure 6**, **Supplementary Table S2**). Conversely, β-lactam resistance-associated pathways decreased by 35.4% (*P* = 0.03), suggesting reduced antibiotic resistance potential.

## 5 Discussion

Our analysis revealed a distinct gut microbial signature in children with AR-FC comorbidity, characterized by reduced α-diversity (*P* = 0.003) and significant β-diversity separation (PERMANOVA, *P* = 0.002).A depletion of key SCFA-producing taxa, including *Faecalibacterium prausnitzii* and *Bacteroides stercoris*, was observed alongside an increase in *Bifidobacterium* abundance. (**Figures 2B**, **3B**). These findings are consistent with previous studies associating *Faecalibacterium* depletion with impaired mucosal barrier integrity and Th2-driven inflammatory responses in isolated AR or FC (3, 10). Furthermore, the significant separation in β-diversity (PERMANOVA, *P* = 0.002) between the AR-FC and HC groups underscores that the comorbidity is associated with a distinct overall gut ecosystem structure, extending beyond the depletion of individual taxa.

The observed depletion of SCFA-producing taxa aligns with established mechanisms of immune dysregulation. Smith et al. (14) demonstrated that microbial-derived SCFAs regulate colonic Treg homeostasis via histone deacetylase (HDAC) inhibition, facilitating the expansion of Foxp^3+^ Treg populations. This finding provides mechanistic validation for the inferred immune dysregulation in our cohort, where depletion of SCFA-producing taxa such as *Faecalibacterium prausnitzii* may be linked to impaired immune tolerance, as supported by established roles of SCFAs in Treg biology (14). Collectively, the observed depletion of SCFA-producing taxa and the associated functional pathway alterations in our cohort are consistent with the proposed microbiota–SCFA–immune axis paradigm (9, 10, 14) in the pathophysiology of AR-FC, though causal relationships require further validation. Additionally, the observed enrichment of *Bifidobacterium*(Log_2_FC = +1.5, *P* = 0.018) in AR-FC subjects at baseline, despite its conventional association with constipation relief (1), raises critical questions about strain-specific effects. For example, *Bifidobacterium longum* BB536 has been shown to enhance cell-mediated immunity (15), whereas other *Bifidobacterium* strains may exert divergent effects under Th2-dominant inflammatory conditions (16). Such heterogeneity highlights the limitation of taxonomic generalization and underscores the need for strain-level resolution when designing probiotic interventions aimed at restoring both gastrointestinal and immune homeostasis.

Functional prediction analysis revealed a distinct metabolic profile in the AR-FC group, characterized by upregulated proteasome activity (*P* = 0.01) and suppressed LPS biosynthesis (*P* = 0.02). The increased proteasome activity may enhance luminal antigen processing, potentially promoting IgE sensitization—a hallmark of AR pathophysiology (7). Conversely, suppressed LPS biosynthesis (*P* = 0.02) may reflect impaired microbial stimulation of innate immunity, potentially reducing TLR4-mediated immune priming and predisposing children to pathogen colonization and chronic inflammation (5, 9). An intriguing finding of our study was the significant upregulation of microbial butanoate and propanoate metabolism pathways in the AR-FC group, as predicted by PICRUSt2, despite a marked depletion of classic SCFA-producing taxa such as *Faecalibacterium prausnitzii*. This apparent paradox may be explained by several non-mutually exclusive mechanisms. First, microbial community resilience may drive residual taxa to upregulate SCFA-biosynthesis genes in a compensatory manner to maintain metabolic homeostasis—though such efforts may be insufficient to offset the functional loss of high-efficiency SCFA producers. Second, the observed pathway enrichment may not translate into increased SCFA output due to post-transcriptional regulation, limited substrate availability, or disruption of cross-feeding networks essential for efficient SCFA synthesis. Such decoupling between genetic potential and actual metabolic flux has been documented in other dysbiotic conditions. Thus, the functional upregulation may reflect a futile compensatory response or a state of metabolic inefficiency, rather than true SCFA sufficiency. This interpretation, however, requires validation through direct metabolomic quantification of SCFAs in future studies. These findings collectively suggest that the gut microbiota may serve as a potential modulator of the “gut-immune-nose axis,” where dysbiosis is associated with disruptions in both mucosal and systemic immune homeostasis.

The 3-month synbiotic intervention did not induce significant changes in α- or β-diversity. However, several key taxonomic shifts were observed that were in the opposite direction to those seen when comparing HC with AR-FC patients. These included a 54.8% increase in *Faecalibacterium* abundance (*P* < 0.05; **Figure 5C**) and an 85.2% decrease in *Bifidobacterium* abundance. This finding is consistent with the established role of *Faecalibacterium prausnitzii* as a primary butyrate producer, which enhances mucosal barrier integrity and promotes intestinal motility through SCFA-mediated mechanisms (14, 17). Although the current study did not directly quantify fecal SCFA levels, the observed *Faecalibacterium* enrichment, coupled with constipation relief, supports the hypothesis that synbiotic-induced microbial remodeling contributes to functional improvement. In a related study, Erhardt et al. (18) reported that a prebiotic intervention similarly increased fecal SCFA levels and enriched beneficial taxa including *Bifidobacterium* in subjects with functional constipation, reinforcing the role of fiber-driven microbial modulation in gastrointestinal health. However, the present study extends these observations by demonstrating a direct association between *Faecalibacterium* expansion and clinical symptom improvement in children with AR-FC comorbidity.

Our investigation identified a pronounced 85.2% reduction in *Bifidobacterium* abundance following synbiotic intervention. This reproducible pattern across distinct clinical cohorts (19) suggests that diminished *Bifidobacterium* levels may represent a potential microbial signature of effective constipation relief. The pronounced reduction in *Bifidobacterium* (−85.2%) may reflect ecological niche competition, as Faecalibacterium and other fiber-fermenting taxa outcompete carbohydrate-preferring *Bifidobacteria* under high-fiber conditions (20). Although **Figure 5D** illustrates the post-intervention decline in *Bifidobacterium*, direct competitive interactions warrant further validation. This trade-off aligns with findings by Scott et al. (20), who demonstrated that *Bifidobacterium adolescentis* preferentially utilizes fructooligosaccharides over complex fibers. Such strain-specific substrate specialization underscores the importance of developing precision synbiotic formulations that selectively enrich beneficial taxa while preserving microbial ecosystem stability.

Notably, the intervention decreased *Escherichia–Shigella*—a pathobiont linked to intestinal inflammation—by 12.3%, potentially through competitive exclusion mediated by *Lactobacillus* strains via bacteriocin production (21). Concomitantly, we observed a functional transition toward enhanced sulfur metabolism (e.g., an 83.2% increase in assimilatory sulfate reduction; **Figure 6**) and a 35.4% reduction in β-lactam resistance pathways. While these metabolic shifts may reflect microbial adaptation to the synbiotic regime, their direct contribution to clinical symptom improvement remains speculative. Enhanced sulfur assimilation has been previously associated with improved redox homeostasis and mucosal protection (22), whereas the decrease in antibiotic resistance genes may indicate a reduction in potential pathogen load or a shift in microbial community structure under dietary modulation (23). Although prior studies have emphasized butyrate-centered mechanisms in microbiome-targeted therapies for AR (16), our synbiotic intervention appears to have induced a broader spectrum of metabolic changes beyond SCFA production. These functional changes, while mechanistically intriguing, should be interpreted with caution and validated in future studies incorporating metabolomic profiling and larger cohorts.

While the integrated taxonomic, functional, and interventional framework of this study provides valuable mechanistic insights, several limitations should be acknowledged. First, functional prediction analysis were inferred from 16S rRNA gene data using PICRUSt2, an approach that remains inherently predictive and hypothesis-generating rather than confirmatory. The absence of direct SCFA quantification via metabolomic techniques (e.g., LC–MS) represents a key limitation; therefore, future investigations should incorporate targeted metabolomic validation to substantiate the inferred pathway alterations. Second, the cross-sectional study design constrains causal interpretation, underscoring the need for longitudinal analyses to monitor microbiota dynamics throughout AR-FC progression. Third, the relatively small intervention cohort (n = 13) and restricted age range (3–7 years) limit generalizability and reduce statistical power to detect subtle phenotypic effects. Future studies should integrate multi-site cohorts and combine taxonomic, functional, and interventional profiling to comprehensively elucidate the microbiota–SCFA–immune axis in AR-FC comorbidity. Parallel characterization of the nasal (BALT-associated) and intestinal microbiota could delineate site-specific microbial signatures and their roles in disease interplay. Moreover, quantifying mucosal immune mediators—such as secretory IgA (SIgA) and local cytokine networks—in nasal and intestinal secretions would provide essential functional evidence of immune crosstalk (24, 25). Although beyond the current study’s scope, such integrated immuno-metagenomic approaches are crucial to mechanistically link microbial alterations with host immune regulation.

Translational research should prioritize interventions that selectively promote the proliferation and functional activity of *Faecalibacterium*, potentially through synergistic probiotic formulations (e.g., *Lactobacillus rhamnosus* GG, known to foster a supportive microbial niche) combined with prebiotic substrates to augment SCFA biosynthesis while mitigating Th2-skewing immune responses. Preclinical validation using germ-free murine models colonized with AR-FC-derived microbiota could delineate causal relationships between *Faecalibacterium* depletion and nasal hypersensitivity. Future therapeutic strategies could explore optimized SCFAs delivery approaches to enhance local bioavailability in the colon, building upon established roles of butyrate in mucosal immunity (14, 17). However, butyrate may not be the sole mediator of the observed effects; other SCFAs (e.g., propionate, acetate), shifts in sulfur metabolism, or competitive exclusion of pathobionts by probiotic strains may also contribute to the clinical outcomes (20, 21).

## 6 Conclusion

This study suggests that gut dysbiosis may represent a potential contributor to pediatric AR-FC comorbidity, marked by the depletion of SCFA-producing taxa (e.g., Faecalibacterium prausnitzii and Bacteroides stercoris) and functional shifts favoring proteasome activity over LPS biosynthesis, a pathway implicated in innate immune regulation. The synbiotic regimen induced taxonomic shifts, including enrichment of Faecalibacterium, which corresponded with constipation alleviation. Conversely, the paradoxical reduction in Bifidobacterium likely reflects competitive substrate utilization, positioning its depletion as a potential biomarker for successful intervention. Functional remodeling post-treatment—including enhanced sulfur metabolism and suppression of antibiotic resistance pathways—demonstrates the adaptive plasticity of gut microbiota under dietary modulation. These alterations implicate disrupted microbial-immune crosstalk along the microbiota–SCFA–immune axis. Translationally, the findings support a dual therapeutic framework: (1) restoration of SCFA-mediated mucosal immunity through targeted synbiotic formulations, and (2) optimization of dietary fiber composition to maintain commensal equilibrium and minimize ecological disruption. Collectively, these findings refine the mechanistic understanding of the microbiota–SCFA–immune axis and provide a foundation for precision microbiome-based therapeutics in pediatric AR-FC provides a well-rounded close linking mechanisms, translational value, and future potential.

Despite these advances, certain limitations, including the small cohort size and reliance on *16S rRNA*-based functional inference, necessitate validation through longitudinal, metagenomic, and metabolomic studies. Future interventions should emphasize strain-specific synbiotic formulations designed to promote *Faecalibacterium* expansion while preserving commensal *Bifidobacterium*.

## Data Availability

The data presented in the study are deposited in the NCBI Sequence Archive (SRA) database repository, accession number is PRJNA1098454. URL: https://dataview.ncbi.nlm.nih.gov/object/PRJNA1098454?reviewer=4cijm90t0u9btm1plm1c7md86m.

## Supplementary material

Supplementary Figure 1 Functional prediction of altered microbial metabolic pathways in children with AR-FC compared to healthy controls (HC) at baseline. The figure displays KEGG pathways that were differentially abundant between the AR-FC and HC groups, as predicted by PICRUSt2 analysis of 16S rRNA gene sequencing data. Pathway enrichment and depletion were assessed using the Wilcoxon rank-sum test with false discovery rate (FDR) correction (adjusted *P* < 0.05). Pathways related to SCFA biosynthesis, including butanoate and propanoate metabolism (both significantly upregulated in AR-FC, FDR-adjusted *P* < 0.01), are indicated. The asterisks denote statistical significance based on FDR-adjusted *P*-values: **P* < 0.05, ***P* < 0.01, ****P* < 0.001.
