# Supplementary material for: Gut microbiota-driven dysbiosis of the SCFA-immune axis in pediatric allergic rhinitis-constipation comorbidity: mechanisms and synbiotic remodeling
Source: Front Immunol. 2025 Dec 3;16:1639359. doi: 10.3389/fimmu.2025.1639359 (PMC12708522; doi:10.3389/fimmu.2025.1639359)
Supplement: Supplementary Figure 1 — Functional prediction of altered microbial metabolic pathways in children with AR-FC compared to healthy controls (HC) at baseline. The figure displays KEGG pathways that were differentially abundant between the AR-FC and HC groups, as predicted by PICRUSt2 analysis of 16S rRNA gene sequencing data. Pathway enrichment and depletion were assessed using the Wilcoxon rank-sum test with false discovery rate (FDR) correction (adjusted P < 0.05). Pathways related to SCFA biosynthesis, including butanoate and propanoate metabolism (both significantly upregulated in AR-FC, FDR-adjusted P < 0.01), are indicated. The asterisks denote statistical significance based on FDR-adjusted P-values: *P < 0.05, **P < 0.01, ***P < 0.001. [file SupplementaryFile1.docx]

Supplementary Material

1. **Light field measurement of the room in which the triplefins were housed**  **Page**

- Figure S1 2
- Figure S2 3

1. **Fluorescence of S. porcus**

- Figure S3 4

1. **S. porcus movement**

- Figure S4 5
- Figure S5 5

**1. Light field in the room in which the triplefins were housed**


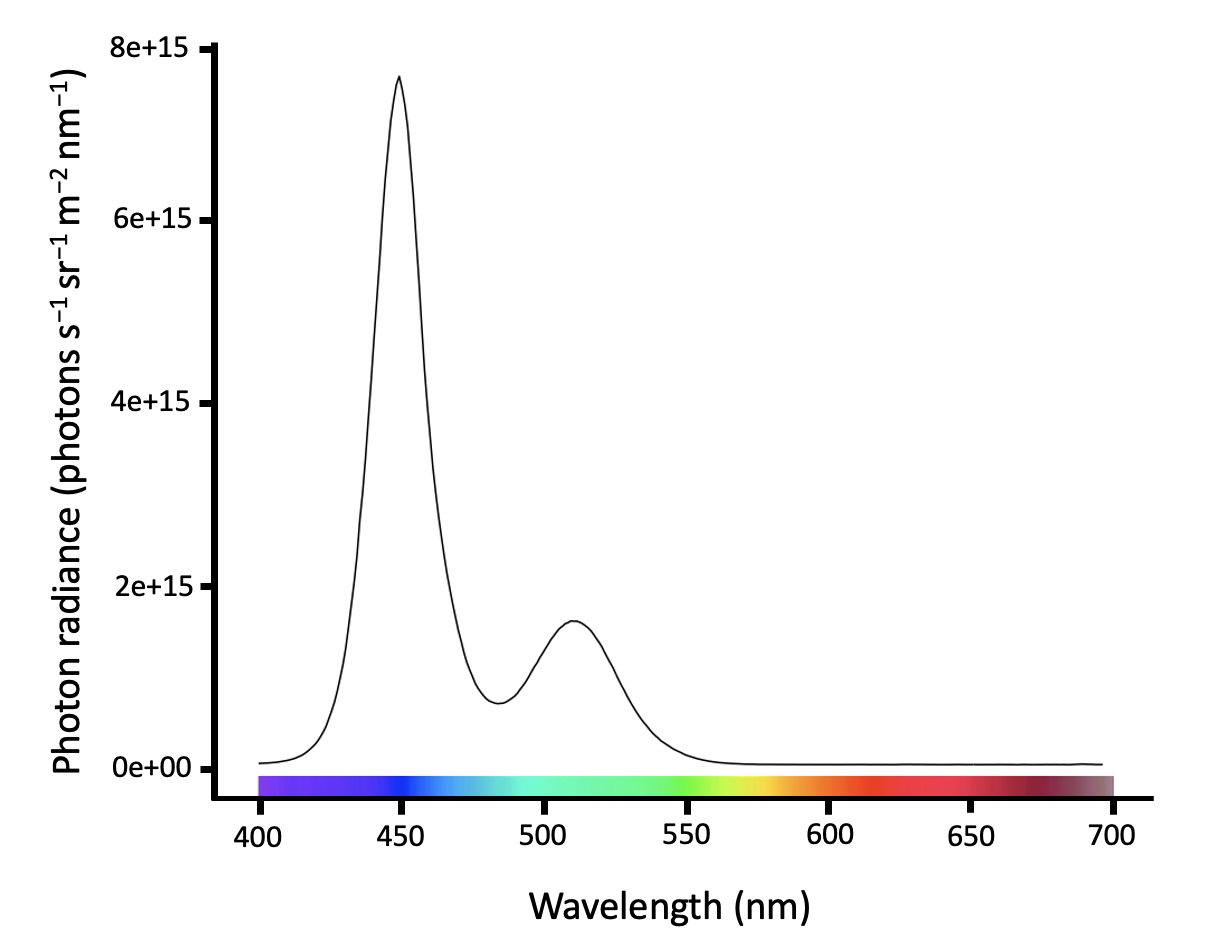


**Figure S1: Light field in the room in which triplefins were housed (Y-axis linear).**

Photon radiance of a diffuse white standard (PTFE) taken with a SpectraScan® PR-740 spectroradiometer.


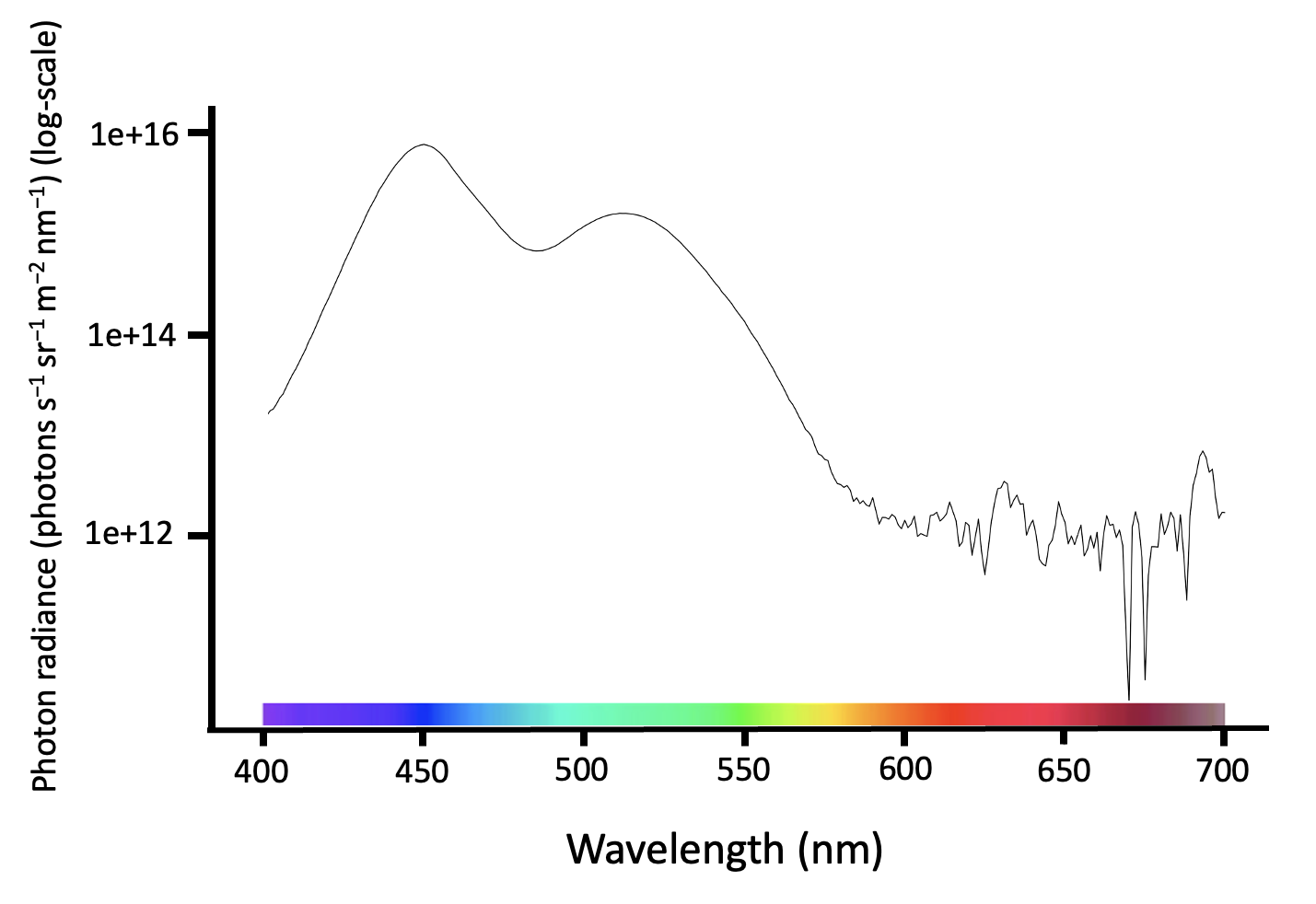


**Figure S2: Light field in the room in which triplefins were housed (Y-axis log_10_ transformed).** Photon radiance of a diffuse white standard (PTFE) taken with a SpectraScan® PR-740 spectroradiometer. Same data as in Figure S1, log transformed.

**2. *S. porcus* red fluorescence**


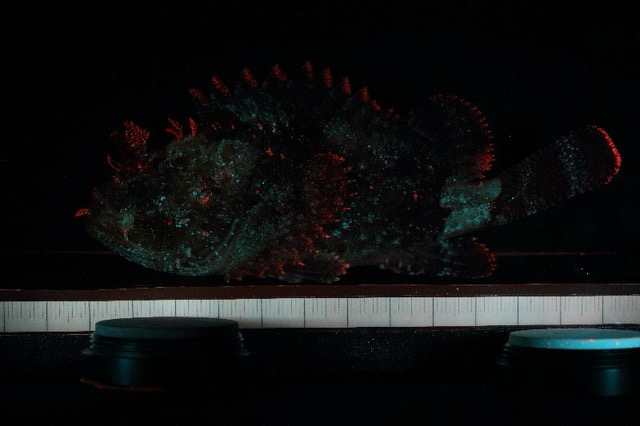


**Figure S3: Photo of *S. Porcus* photographed in the lab under strong blue LED light as seen through a yellow filter to block the excitation light.** The red fluorescence of a *S. porcus* is limited and weak in comparison to other scorpionfish species (Anthes et al. 2026) (picture by Jenny Theobald)

**3. *S. porcus* movement**


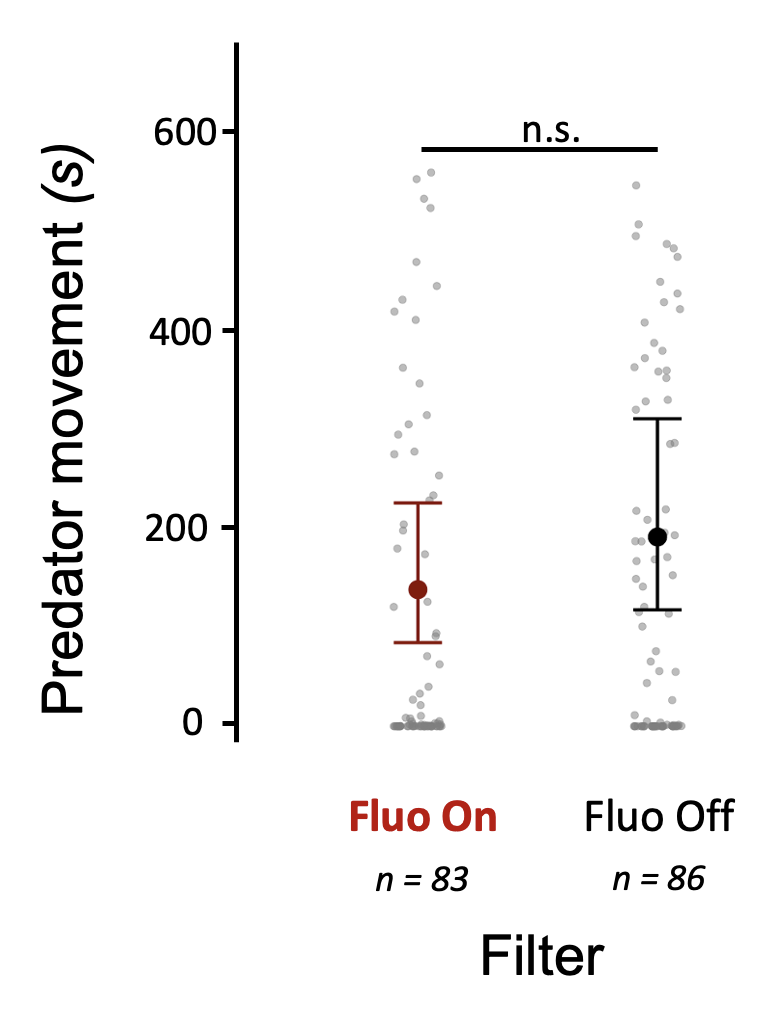


**Figure S4: Effect of the different filters on the movement of a *S. porcus.***

There is no correlation between the different filters and the total time (seconds) that a black scorpionfish (*S. porcus*) was moving during a run.


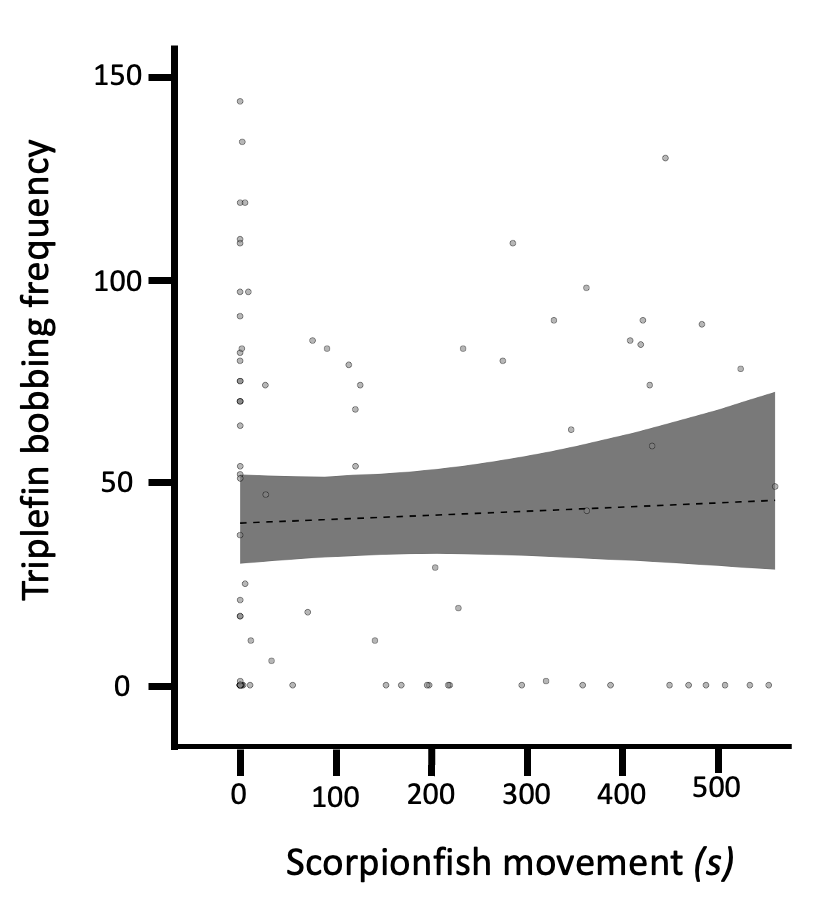


**Figure S5: Effect of scorpionfish movement on the total number of bobs of a triplefin during a run.**

There is no correlation between scorpionfish movement and the number of bobs a triplefin performs. Dashed line indicates the model predictions, and the grey error zone represents the 95% compatibility intervals (CI) (see also Fig. S4)
